# Focus distance estimation from photographed faces: a test of *PerspectiveX* using 1709 frontal and profile photographs from DSLR and smartphone cameras

**DOI:** 10.1007/s00414-023-03078-y

**Published:** 2023-09-13

**Authors:** Sean S. Healy, Carl N. Stephan

**Affiliations:** Laboratory for Human Craniofacial and Skeletal Identification (HuCS-ID Lab), School of Biomedical Sciences, The University of Queensland, Brisbane, 4072 Australia

**Keywords:** Forensic anthropology, Photographic superimposition, Video superimposition, Lens, Focal length, Subject-to-camera distance, Photography

## Abstract

As focus distance (FD) sets perspective, it is an important consideration for the forensic analysis of faces in photographs, including those used for craniofacial superimposition. In the craniofacial superimposition domain, the *PerspectiveX* algorithm has been suggested for FD estimation. This algorithm uses a mean value of palpebral fissure length, as a scale, to estimate the FD. So far, *PerspectiveX* has not been validated for profile view photographs or for photographs taken with smartphones. This study tests *PerspectiveX* in both front and profile views, using multiple DSLR cameras, lenses and smartphones. In total, 1709 frontal and 1709 profile photographs of 10 adult participants were tested at 15 ground truth FDs using three DSLR cameras with 12 camera/lens combinations, five smartphone back cameras and four smartphone front cameras. Across all distances, *PerspectiveX* performed with a mean absolute error (MAE) of 11% and 12% for DSLR photographs in frontal and profile views, respectively, while errors doubled for frontal and profile photographs from smartphones (26% and 27%, respectively). This reverifies FD estimation for frontal DSLR photographs, validates FD estimates from profile view DSLR photographs and shows that FD estimation is currently inaccurate for smartphones. Until such time that FD estimations for facial photographs taken using smartphones improves, DSLR or 35 mm film images should continue to be sought for craniofacial superimpositions.

**Supplementary Information:**

The online version contains supplementary material available at 10.1007/s00414-023-03078-y.

## Introduction

Antemortem face photographs are commonly used to assist forensic identification in a multitude of forensic science contexts [1]. This is due to the highly discriminative nature of faces (and teeth) [2], often allowing positive or negative matches to be determined when face anatomy is compared against suitable reference samples. How facial photographs are used for identification depends on the context of the case. For example, facial photographs may be used to identify perpetrators of crime(s) [3], or to identify victims of crime and decedents [4, 5]. In the former context, forensic facial comparison [6–9] and automated facial recognition [10–12] see frequent use. For decedent identification, facial photographs are often employed to evaluate the anatomical fit of a skull to a known face using a method known as craniofacial superimposition [13–30]. If no anatomical correspondence is found at image comparison, then the remains may be excluded as a plausible candidate for a match, or if correspondence is detected, then further investigation of identity using other methods is warranted [13–30]. This sees craniofacial superimposition often used as a screening test and mostly for exclusions in present day forensic casework [21, 31, 32]. In forensic odontology, dental remains have been used in a similar fashion with face photographs, and this approach is in-turn labelled dental superimposition [33–35].

As facial photographs are formed by the camera’s projection of a 3D scene to a 2D plane, the camera’s vantage point in 3D space sets the face’s perspective in the photographic recording [36–38]. In the computer science domain, this vantage point is often and equivalently described by the group of factors labelled ‘extrinsic camera parameters’ [36–38]. In many forensic fields, the camera is treated as a static object, with the orientation of the subject in the photograph assessed relative to the camera. In craniofacial superimposition, this applies where the parameters describing the scene are often couched as the head pose (yaw, pitch and roll of the head) and the position of the head in 3D space (x, z position in the camera field of view)—note here that translation along the *y*-axis, the focus distance (FD) is often conveniently discounted because it has been deemed too difficult to estimate [24, 39, 40]. A more inclusive and equally valid view is to frame the face as the static object relative to a mobile camera, such that all of the abovementioned characters (head position, pose and FD) are described by the singularity of the camera vantage point. As a contributor to the camera vantage point, the FD component is notable because it is the sole determinant of perspective distortion when the head is positioned in the centre of the field-of-view of the camera [41–44], and thereby, it should not be ignored in favour of other ‘head position’ factors, as is common place in present-day craniofacial superimposition. Subsequently, we specifically award attention to FD estimation in this paper.

In the broader computer science literature, one proposed solution to the FD problem is the use of perspective correction algorithms. Two major methods of correction have been developed, warping functions [45, 46] and deep learning methods (using generative adversarial networks) [47]. These methods hold limits in the context of craniofacial superimposition. The former is unable to deal with changes caused by occlusion, as warping functions cannot create new information, while the latter generates an image that is no longer a true representation of the evidence, thus making its use in a court setting dubious.

The automated facial recognition field has also encountered challenges dealing with FD, and without viable perspective correction methods in this field, it has been recommend that automated recognition systems should be trained on photographs taken from a range of distances [48]. In forensic facial comparison, a paucity of published methods to specifically address perspective distortions has also been recognised [6]. In this field, assessors are reported to qualitatively assess perspective distortion during their image analysis [6]—a highly subjective approach that may vary between practitioners.

In the craniofacial and dental superimposition domains, the analysis permits unlimited acquisition of comparative postmortem photographs of the skull (and/or teeth) at different vantage points for comparison to the antemortem reference face image. While it is possible to iteratively adjust the FD at photography to match the perspective in the AM reference image by trial and error, this is often a tedious and difficult task. When head pose is also an unknown variable, the large number (6) of degrees of freedom [38] in geometrical space makes the task a challenging one as several million possibilities of camera positions may need to be searched to find the correctly corresponding position. In craniofacial superimposition, additional complexity exists as the two objects being compared are not the same (one is a skull, and the other is a face), such that the difficulty of finding correctly corresponding camera vantage points is further increased. This makes it critical that the FD be set not by iterative trial and error, but by some strategic approach [49–51].

Several computer science methods have been developed with the aim of FD estimation. The most common approach uses facial landmark spacing in photographs and comparison average face exemplars (derived from specific pre-set distances) to try to formulate a distance estimate [52–55]. The major limitation of this method is that an individual’s facial variation interferes with the FD estimation, due to the reliance on average parameters. Deep learning has also been applied to FD estimation; however, the majority of approaches have limited accuracy or have been tested on only very small sample sizes [56–58]. One deep learning approach that has produced impressive estimation results on a larger sample size is FacialSCDnet, generating a reported mean relative error of 2.5% [59]; however, this method is not without limitations. First, the primary training and test images used for FacialSCDnet are synthetic computer-generated 3D photographs made from 3D face scans, with only a limited number of real-world 2D photographs used. Second, the FacialSCDnet neural networks are each presently specific to one of four full-frame equivalent focal lengths of 27, 35, 55 and 85 mm [59] and require the acquisition of new images to train models for focal lengths other than those mentioned above. Lastly, and as applicable to all deep learning approaches, these methods represent ‘black boxes’, such that it is impossible to explain, in a courtroom, how exactly the models work [60].

In contrast to artificial intelligence approaches, the *PerspectiveX* algorithm described in 2017 [49] uses the well-known and straightforward mathematical relationships of similar triangles to estimate the FD [61]. The method draws on the largely invariant nature of humans’ palpebral fissure lengths (standard deviation = 1.2 mm) that enables a sample mean to be used as a known length in lieu of an individual’s exact measurement (the individual in the AM image is now deceased; thus, this soft tissue measurement cannot be acquired for the individual). *PerspectiveX* has been subject to three validation studies using real-world DSLR frontal view photographs, generating mean signed error (MSE) of 6.6% in 2017 [49], 3.8% for prime lenses and 4.4% for zoom lenses in 2021 [50] and 8.0% in 2022 [51]. Conversely, tests of *PerspectiveX* using synthetic photographs are reported to generate 15.3% mean relative error [59]. In 2022, *PerspectiveX* was, for the first time, extended to profile view photographs, producing 7.8% MAE in a pilot study on three subjects, again using DSLR cameras [51].

Three years following *PerspectiveX*’s proposal [49], MediaPipe Iris was released in 2020 [62]. Like *PerspectiveX*, MediaPipe Iris uses an anatomical character to estimate the FD, but rather than using the palpebral fissure length, it uses the iris diameter, measured via a deep learning network, making MediaPipe Iris a fully automated method. The use of the iris diameter was considered for *PerspectiveX* at the time it was developed; however, the palpebral fissure length was preferred due to its larger size and subsequent minimization of the relative technical errors of measurement from the photographs, especially where image resolutions may prevent precise determination of the exact edges of fine structures at long FD [49]. MediaPipe Iris is reported to produce FD estimates with only 2.4% mean relative error [62] when FDs are less than 2 m [62]. The small size of the iris likely limits MediaPipe Iris’s functionality for longer FD as applicable in the craniofacial superimposition context.

In this study, we award attention to the accuracy of FD estimation using the *PerspectiveX* algorithm as relevant to the craniofacial superimposition context where FDs between 0.2 and 10 m are likely to be encountered. In particular, we investigate (1) FD estimation for profile photographs in a large sample of cameras, lenses and subjects and (2) utility of smartphone camera images for FD estimates.

## Methods

### Study 1: *PerspectiveX* error for frontal and profile photographs taking with digital single-lens reflex cameras (DSLRs)

Ten adult participants were photographed at six known distances between 1 and 10 m using three different full-frame DSLR camera bodies: a Canon^®^ EOS 6D, a Canon^®^ EOS 6D Mark II and a Nikon^®^ D780 (Table 1). Each camera was fitted with three fixed prime lenses and a single variable zoom lens, of which the two extreme end-range focal lengths were used for facial photographs increasing the range of instrumentation compared to other studies [49–51] for FD estimation using *PerspectiveX* (Table 2).

**Table 1:** Technical specifications of DSLR cameras used in Study 1

Camera body brand & model		Image sensor dimensions (mm)	Image receptor pixel size (μm)	Resolution (megapixels)
	Canon^®^ EOS^a^ 6D	35.8 × 23.9	6.55	20.2
	Canon^®^ EOS^a^ 6D Mark II	36 × 24	5.67	26.2
	Nikon^®^ D780	35.9 × 23.9	5.90	24.5

^a^Electro-optical system

**Table 2:** Technical specifications and images of DSLR lenses used in the Study 1

Lens								
Image	Brand	Focus	Focal length (mm)	Aperture	Other Differentiators	Type	Focal Lengths used (mm)	Paired to camera(s)
	Canon^®^	EF^a^	50	f/1.8	Generation II	Prime	50	Canon^®^ 6D & Canon^®^ 6D Mark II
	Canon^®^	EF^a^	85	f/1.8	USM^b^	Prime	85	Canon^®^ 6D & Canon^®^ 6D Mark II
	Canon^®^	Macro EF^a^	100	f/2.8	L-type	Prime	100	Canon^®^ 6D & Canon^®^ 6D Mark II
	Canon^®^	EF^a^	24-105	f/1.8	USM^b^	Zoom	24 & 105	Canon^®^ 6D & Canon^®^ 6D Mark II
	Nikon^®^	EF^a^	50	f/1.8	G-type	Prime	50	Nikon^®^ D780
	Nikon^®^	AF-S^c^	85	f/1.8	G-type	Prime	85	Nikon^®^ D780
	Nikon^®^	AF-S^c^	105	f/2.8	G-type IF^d^ ED^e^	Prime	105	Nikon^®^ D780
	Nikon^®^	AF-S^c^	24-120	f/4	G-type ED^e^ VR^f^	Zoom	24 & 120	Nikon^®^ D780

^a^Electro-focus

^b^Ultrasonic motor

^c^Auto focus with silent wave motor

^d^Internal focusing

^e^Extra-low dispersion glass

^f^Vibration reduction

**Fig. 1:**
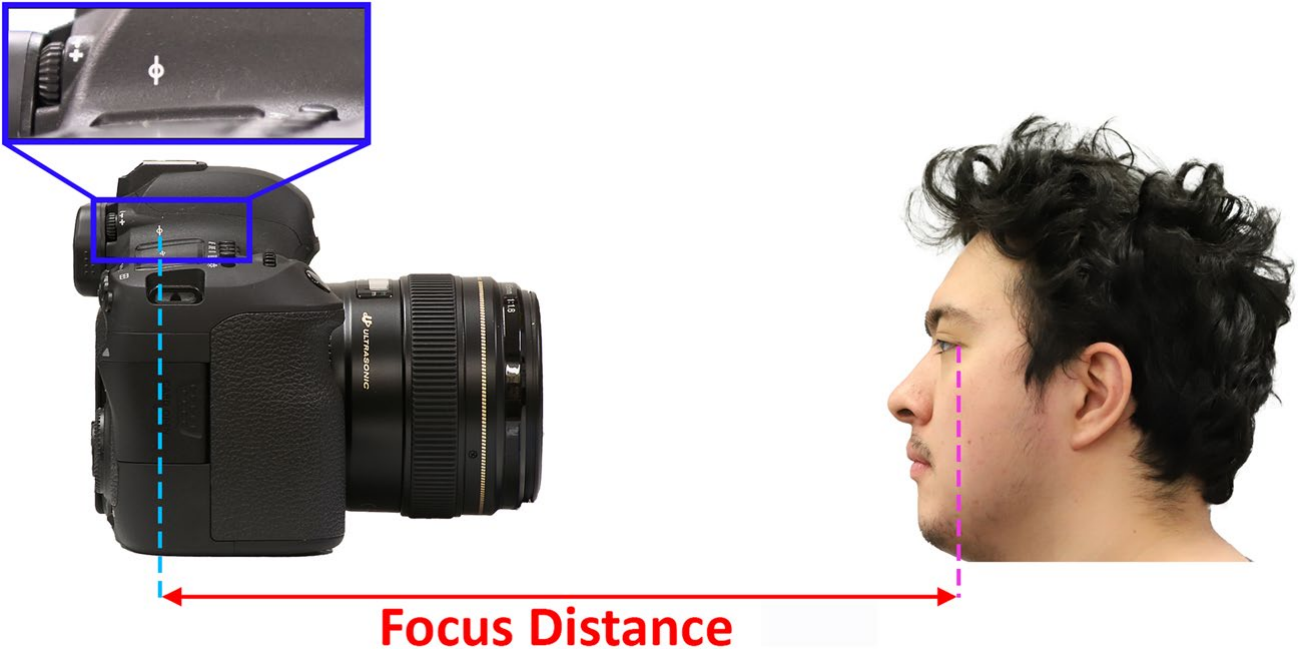
The FD is the length between the image receptor plane, marked by the focal plane indicator (ϕ) on the camera housing (blue-dotted line) and exocanthion (purple-dotted line)

Prior to photography, participants were asked to remove any headwear or eyewear that obscured pertinent mid-facial features and were seated on a chair. The required FDs were measured with a Nicholson^®^ fibreglass spool tape with the tapes end placed in line with the participant’s palpebral fissure when the head was facing forwards towards the camera (Fig. 1). The camera was mounted on a Vanguard^®^ Alta Pro series 263AT and set to a height level with the participant’s eyes/head when seated, such that the participant was in the centre of the camera’s field of view. Photography then commenced, with the camera starting at the 1 m mark.

**Fig. 2:**
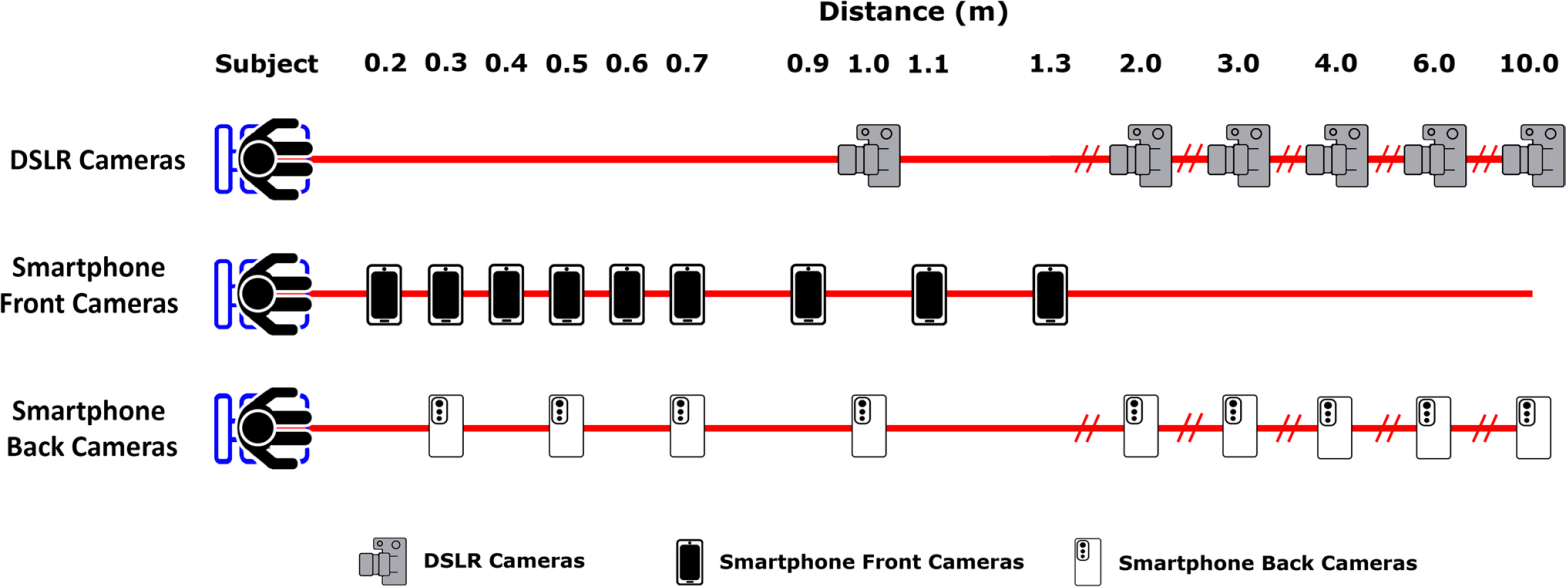
Focus distances used in Study 1 and 2 for frontal and profile photographs taken using DSLR, smartphone front and smartphone back cameras

All cameras were set to manual mode, with the following settings: ISO640, f/13, a shutter speed of six, no flash, a 2 s shutter delay (to avoid any camera shake) and manual camera focusing. At each distance, with each focal length, participants were photographed four times, twice in frontal and twice profile view to protect against recordings of blinking in images and such that the best image could be selected for analysis. During the first two photographs, the participant was asked to look directly at the lens with a closed mouth and neutral expression, while in the last two photographs, the participant was instructed to stare at a fixed mark on a wall, producing 42° head rotation to the right. Five sets of four photographs are taken at each distance with each camera—one for each fixed prime lens and two for each variable zoom lens, the latter possessing a set for both the minimum and maximum focal length of the variable adjustment lens. After all photographs for an FD were taken, the camera was moved to the next FD position, where the process was repeated (Fig. 2), yielding in total 120 photographs for any given camera body. Once all 120 photographs for a camera were obtained, the camera body was changed, and the process was repeated for the next camera body, generating a total of 360 photographs per participant (Table 3).

**Table 3:** Photographs required at each distance for each lens and camera combinations. (NB. The set of 4 photographs at each FD includes duplicate images to protect against blinking and/or incorrect subject position)

					Focus distances (m)					
Camera	Lens			Focal length (mm)	1	2	3	4	6	10
	Brand	Details	Type							
Canon^®^ 6D	Canon^®^	EF f/1.8 II	Prime	50	4	4	4	4	4	4
	Canon^®^	EF f/1.8 USM	Prime	85	4	4	4	4	4	4
	Canon^®^	Macro EF f/2.8L	Prime	100	4	4	4	4	4	4
	Canon^®^	EF 24–105 mm f/4L USM	Zoom	24	4	4	4	4	4	4
				105	4	4	4	4	4	4
Canon^®^ EOS 6D Mark II	Canon^®^	EF f/1.8 II	Prime	50	4	4	4	4	4	4
	Canon^®^	EF f/1.8 USM	Prime	85	4	4	4	4	4	4
	Canon^®^	Macro EF f/2.8L	Prime	100	4	4	4	4	4	4
	Canon^®^	EF 24105 mm f/4L USM	Zoom	24	4	4	4	4	4	4
				105	4	4	4	4	4	4
Nikon^®^ D780	Nikon^®^	AF-S NIKKOR f/1.8G	Prime	50	4	4	4	4	4	4
	Nikon^®^	AF-S NIKKOR f/1.8G	Prime	85	4	4	4	4	4	4
	Nikon^®^	AF-S VR Micro NIKKOR f/2,8G IF ED	Prime	105	4	4	4	4	4	4
	Nikon^®^	AF-S NIKKOR 24–120 mm f/4G ED VR	Zoom	24	4	4	4	4	4	4
				120	4	4	4	4	4	4

### Study 2: *PerspectiveX* errors for frontal and profile photographs taking with smartphone cameras

The 10 participants were additionally photographed with five smartphones, using the rear camera of all the devices and the front cameras of four (Table 4 and Fig. 2). NB. The iPhone™ XR front camera was omitted due to the absence of its technical specifications in manufacturer descriptions/other sources.

**Table 4:** Technical specifications of smartphone cameras used in Study 2

Phone		Camera	Focal length (mm)	Image receptor pixel size (μm)	Resolution (megapixels)
	Apple^®^ iPhone™ 5	Back	4.15	1.4	8
		Front	2.18	1.8	1.2
	Apple^®^ iPhone™ XR	Back	4.25	1.4	12
	Motorola^®^ Moto G9 Plus	Back	5.53	1.6	48
		Front	3.78	2.0	20
	Oppo^®^ A57	Back	3.46	1.1	13
		Front	3.57	1.0	16
	Samsung^®^ Galaxy A31	Back	4.60	1.6	64
		Front	3.80	2.0	16

A custom-built gantry containing 10 Joby^®^ GripTight™ ONE smartphone mounts at pre-set distances (Fig. S1) allowed for all front smartphone camera photographs to be taken, while the tripod remained in a position 0.8 m from the participant. During photography, smartphone cameras were set to auto mode, with autofocus enabled, to emulate the ‘point and click’ nature of most smartphone photographs. A Zttopo^®^ wireless Bluetooth camera remote shutter was used to remotely activate the shutter release of the smartphone cameras, avoiding any motion blur caused by camera shake. Each of the smartphone front cameras was used to take four photographs, following the same procedure as for the DSLR cameras, at the distances described in Table 5. Photographs were taken using the smartphone back cameras at the distances described in Table 5, following the same photography procedure (Fig. 2). The distances between 2 and 10 m were achieved by placing a single Joby^®^ GripTight^™^ ONE mount on a second tripod (Vanguard^®^ Alta Pro series 263AT) and positioning it at each distance with a plumb line against the spool tape. All photographs in both studies were acquired in .jpeg format and were taken by first author (SH).

**Table 5:** Photographs taken at each distance for smartphone front and back cameras. The set of 4 photographs at each distance includes the duplicates

		Focus distance (m)													
Phone	Camera	0.2	0.3	0.4	0.5	0.6	0.7	0.9	1	1.1	1.3	2	4	6	10
Apple^®^ iPhone™ 5	Front	4	4	4	4	4	4	4	-	4	4	-	-	-	-
	Back	-	4	-	4	-	4	-	4	-	-	4	4	4	4
Apple^®^ iPhone™ XR	Back	-	4	-	4	-	4	-	4	-	-	4	4	4	4
Motorola^®^ Moto G9 Plus	Front	4	4	4	4	4	4	4	-	4	4	-	-	-	-
	Back	-	4	-	4	-	4	-	4	-	-	4	4	4	4
Oppo^®^ A57	Front	4	4	4	4	4	4	4	-	4	4	-	-	-	-
	Back	-	4	-	4	-	4	-	4	-	-	4	4	4	4
Samsung^®^ Galaxy A31	Front	4	4	4	4	4	4	4	-	4	4	-	-	-	-
	Back	-	4	-	4	-	4	-	4	-	-	4	4	4	4

### Focus distance estimation

**Fig. 3:**
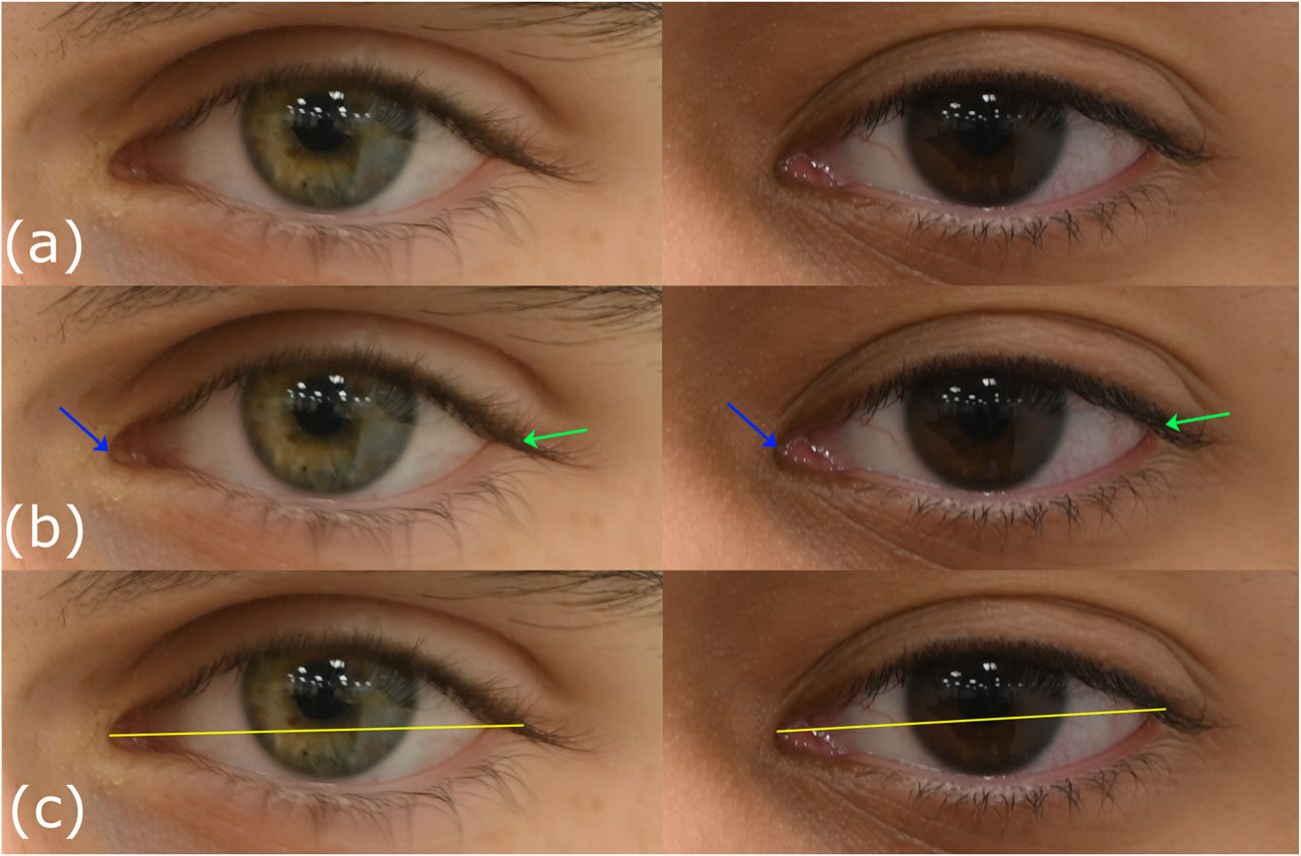
Example palpebral fissure length measurement. Two photographs of participants taken with a Nikon^®^ D780 camera body and a Nikon^®^ AF-S VR Micro NIKKOR 105 mm f/2,8G IF ED lens at a FD of 1 m. Participant 1 is in the left column; participant 2 is in the right column. Row (a) cropped raw images to show the left eye of each participant. Row (b) the same images as (a) but with the endocanthion (blue arrow) and exocanthion (green arrow) marked. Row (c) the yellow line represents the palpebral fissure length measurement between endo- and exocanthion landmarks

**Fig. 4:**
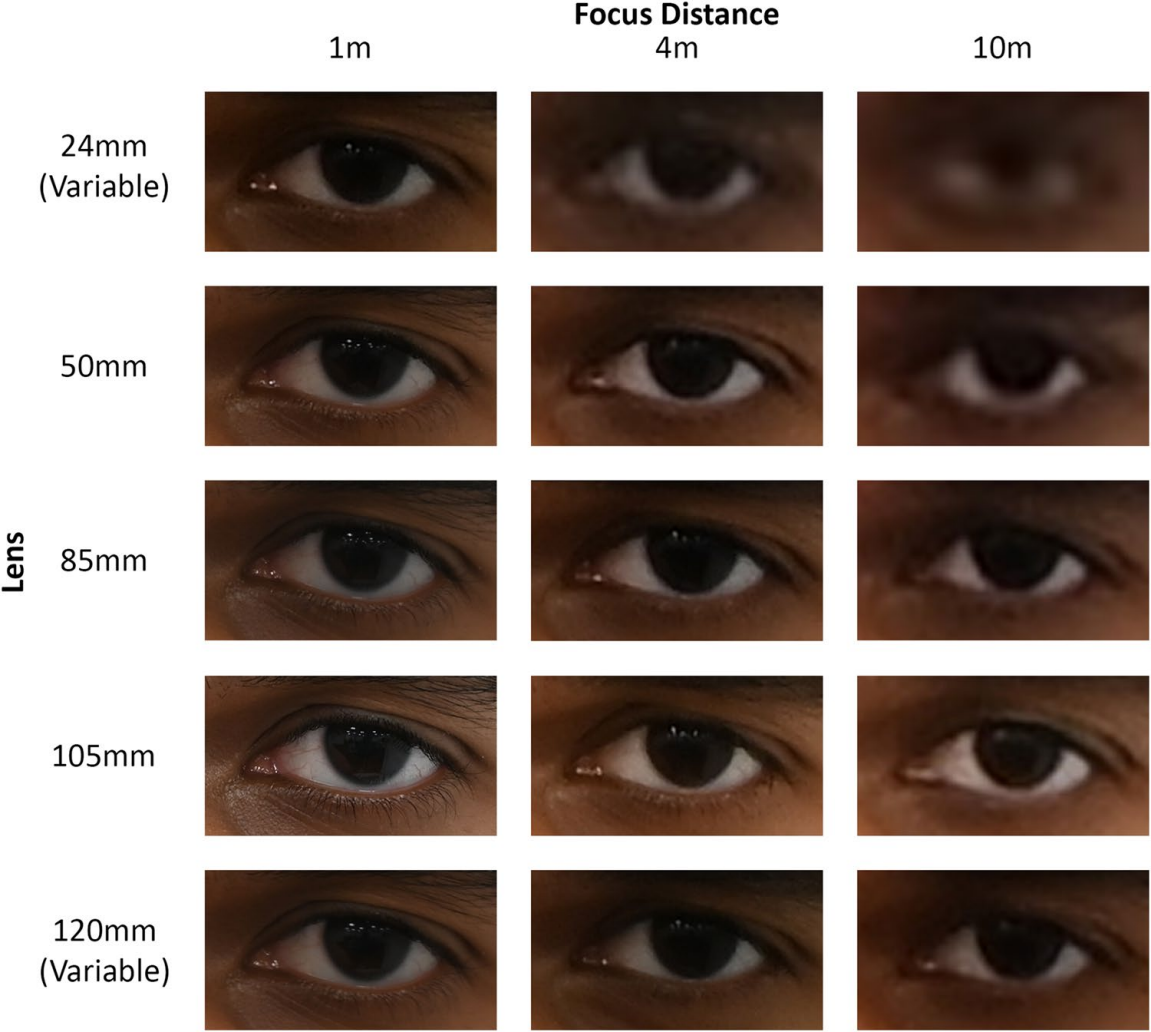
Example palpebral fissure recorded at different FDs and with different camera lenses for the same subject photographed with the same camera body (Nikon^®^ D780). All images have been enlarged to match the 105 mm focal length image at 1 m. Note: this enlargement has resulted in resampling of the lower resolution images to 300 pixels per inch, adding some smoothing to what were originally more pixelated images. For similar figures for other camera bodies/lenses, see Supplementary Online Files: Fig. S2-S5

The best quality photograph of each duplicate pair, and where the subject was not in any stage of blinking, was chosen from the image pair for measurement. *PerspectiveX* was used to estimate the FD for all 1709 photographs taken in frontal and 1709 photographs taken in profile views. In frontal view photographs, the PFL was measured in Adobe^®^ Photoshop^®^ 2022 using the Photoshop^®^ ruler tool to two decimal places, as per [51] (Fig. 3). In cases where the resolution of the face was low (due to small focus length of the lenses combined with long FD) or the eyelashes obscured the exocanthion, the landmarks were estimated by triangulation using adjacent features (such as curving contour of upper and lower lids) and shape-from-shading information, again after methods of Stephan [51]. Example photographs at different FDs obtained with different cameras and lenses are provided in Fig. 4 and Figure S2–S5. All PFL measurements were undertaken in pixel units by the first author (SH).

**Fig. 5:**
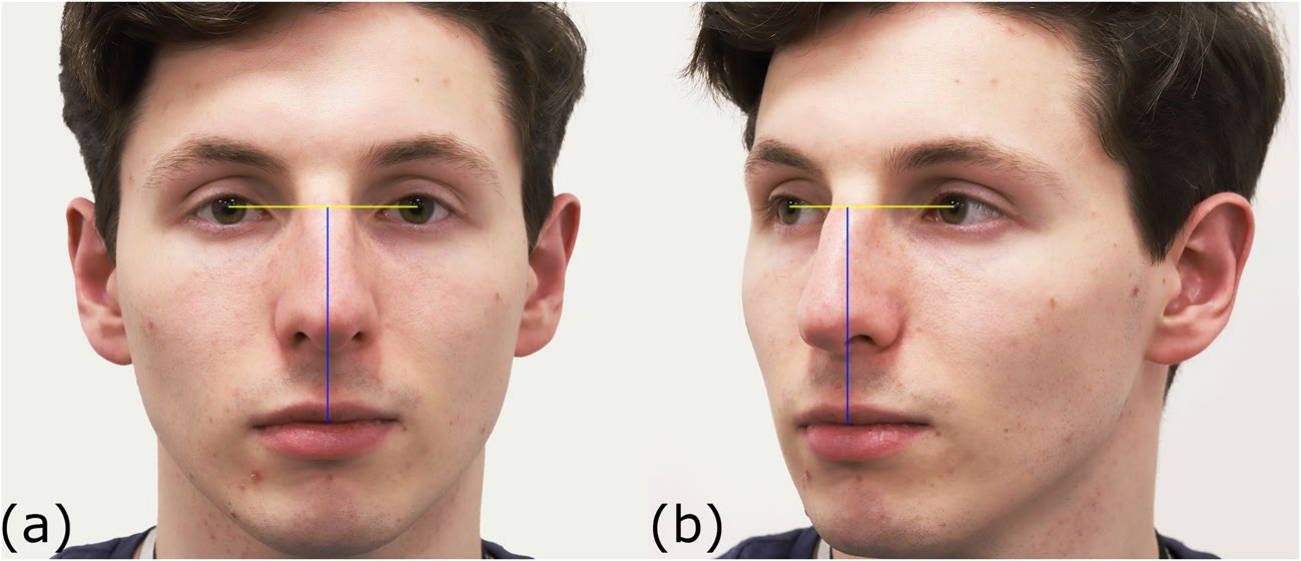
Example pupil chord to stomion measurement. **a** Frontal photograph of taken with a Canon^®^ EOS 6D Mark II camera body and a Canon^®^ macro EF 100 mm f/2.8L prime lens. Ground truth FD is 1 m. Pupil chord is represented as a yellow line. Pupil chord to stomion measurement is represented by a blue line. **b** A partial profile photograph taken at the same distance with the same camera body and lens and with the measurements from **a** again represented

For estimation of the FD from partial profile photographs, the pupil chord to stomion distance was measured in both frontal and profile photographs, after [51, 63] (Fig. 5). *PerspectiveX* was then used to estimate the FD of each frontal photograph using (1) the measured palpebral fissure length from the photograph (x) in pixel units, the focal length of the camera lens (*f*) in millimetre units, (2) the mean palpebral fissure length (A) in millimetre units (according to Farkas [64]) and (3) the manufacturer reported image pixel size of the camera (y) in millimetre units [49]. Whereas the image receptor pixel sizes and focal lengths of the DSLR cameras and lenses were easily determined from the manufacturer website, Exif metadata readers or written on the lenses themselves, the same information was considerably more difficult to acquire for smartphones. It was common for manufacturers to list only 35 mm equivalent focal lengths (not the lens’ actual focal length), and the image receptor pixel sizes were rarely detailed. Due to this, raw focal length values were found either through third party websites (http://explorecams.com) or by installing an application called Device Info HW (deviceinfohw.ru), which listed both values for inputted images.

For profile photographs, an estimate of the real-life size of pupil chord to stomion was first generated from a frontal photograph, using the equations outlined in [51]. The resultant value was then entered into *PerspectiveX*, along with a pixel length of pupil chord-to-stomion distance in the profile photograph, generating a FD estimate [51]. For this study, frontal and profile photograph pairs used for FD estimation were taken at the same FD with the same camera and lens combination.

Eight of the 10 participants had an estimate recorded for every camera/lens and distance combination, while two participants did not, missing photographs for the Oppo^®^ A57 back camera and iPhone™ XR back camera, respectively. Subsequently, these two cameras had a sample size of nine participants rather than 10. Mean signed error, MAE, percent mean absolute error (MAE%) and standard error of the estimate (SEE) were calculated from the difference between the ground truth FD and the estimates. This research was conducted under an institutional ethics approval from the University of Queensland Medicine, Low & Negligible Risk Sub-committee (Approval# 2022/HE000109).

## Results

### Study 1: *PerspectiveX* error from frontal and profile photographs taking with digital single-lens reflex cameras (DSLRs)

**Fig. 6:**
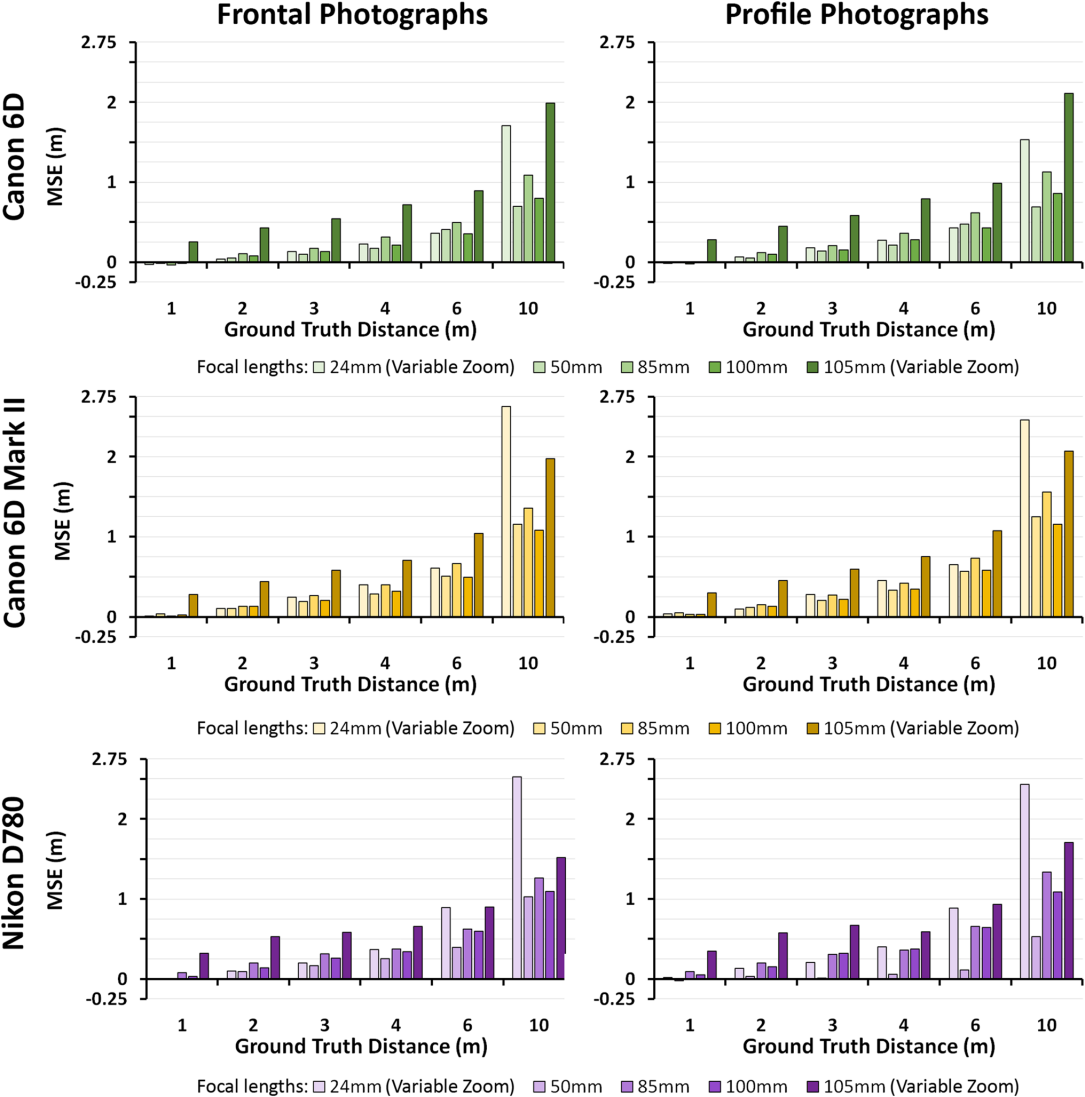
Mean signed error of *PerspectiveX* FD estimations of frontal and profile photographs taken using different DSLR cameras and lenses

*PerspectiveX* was able to estimate the ground truth FDs with limited error for both frontal and profile photographs taken with DSLR cameras, with MSE, MAE and MAE% of 504 mm, 545 mm and 12%, respectively (Fig. 6 and Fig. S6-S7). At short FDs, the error was small (i.e. MSE was ≤ 21 mm at 1 m FD for fixed prime lenses), and it increased smoothly (as expected [49, 50]) for longer FDs in part due to decreasing resolution of the face and ability to measure palpebral fissure length accurately (see, e.g. Fig. 4 for Nikon^®^ D780 and for other cameras: Fig. S2-S5). At distances where the resolution of the face was higher (1–4 m), the error was smaller in magnitude, with MSE, MAE and MAE% of 286 mm, 323 mm and 8%, respectively. Standard errors of the estimate (FD) using *PerspectiveX* are presented in Figure S8, and these show very similar patterns to the mean error plots mentioned above.

The maximum focal length of the variable zoom lens possessed the largest FD estimation errors of all the lenses evaluated, and this was observed across the camera manufacturers (see Fig. 6 for MSE and Fig. S6-S8 for other error statistics). The minimum focal length of the variable zoom lens (24 mm) showed similar error to the fixed prime lens at short FDs (1–4 m) suggesting that antemortem photographs taken with these lenses at short FDs can be used for craniofacial superimpositions (Fig. 6 and Fig. S6-S8). However, practitioners should be alert to the fact that these wide-angle view lenses yielded especially large errors at FDs > 6 m (Fig. S7). There was little difference in performance between camera/lens models or camera/lens manufactures (Fig. 6 and Fig. S6-S8).

Estimation errors in percentage units clearly demonstrate that fixed prime lenses produced consistently small error across the entire FD range and especially within 1–6 m (Fig. S7). The zoom lenses in contrast, generated larger error overall (Fig. S7). The minimum focal length of the variable zoom lens (24 mm) possessed sharply rising error in the 6 to 10 m range. The maximum focal lengths of the variable zoom lens (105 mm for 6D and 6D Mark II and 120 mm for D780) held the largest errors at short FDs (e.g. MAE% = 28% for the Canon 6D at 1 m), which then steadily decreased from 1 to 6 m (e.g. MAE% = 16% for the Canon 6D at 6 m), before increasing in error slightly (e.g. MAE% = 21% for the Canon 6D at 10 m; Fig. S7).

The FD estimates, produced, for the majority of photographs, perspective differences of the face that were less than 1% change in physiognomical facial height—the accepted error tolerance range [41]. There were three cases in which estimates exceeded the 1% facial height limit: 1 m for prime lenses, 1 m for zoom lenses and 2 m for the maximum focal length setting on zoom lens (Table S9).

### Study 2: *PerspectiveX* estimation error for frontal and profile photographs taking with smartphone cameras

**Fig. 7:**
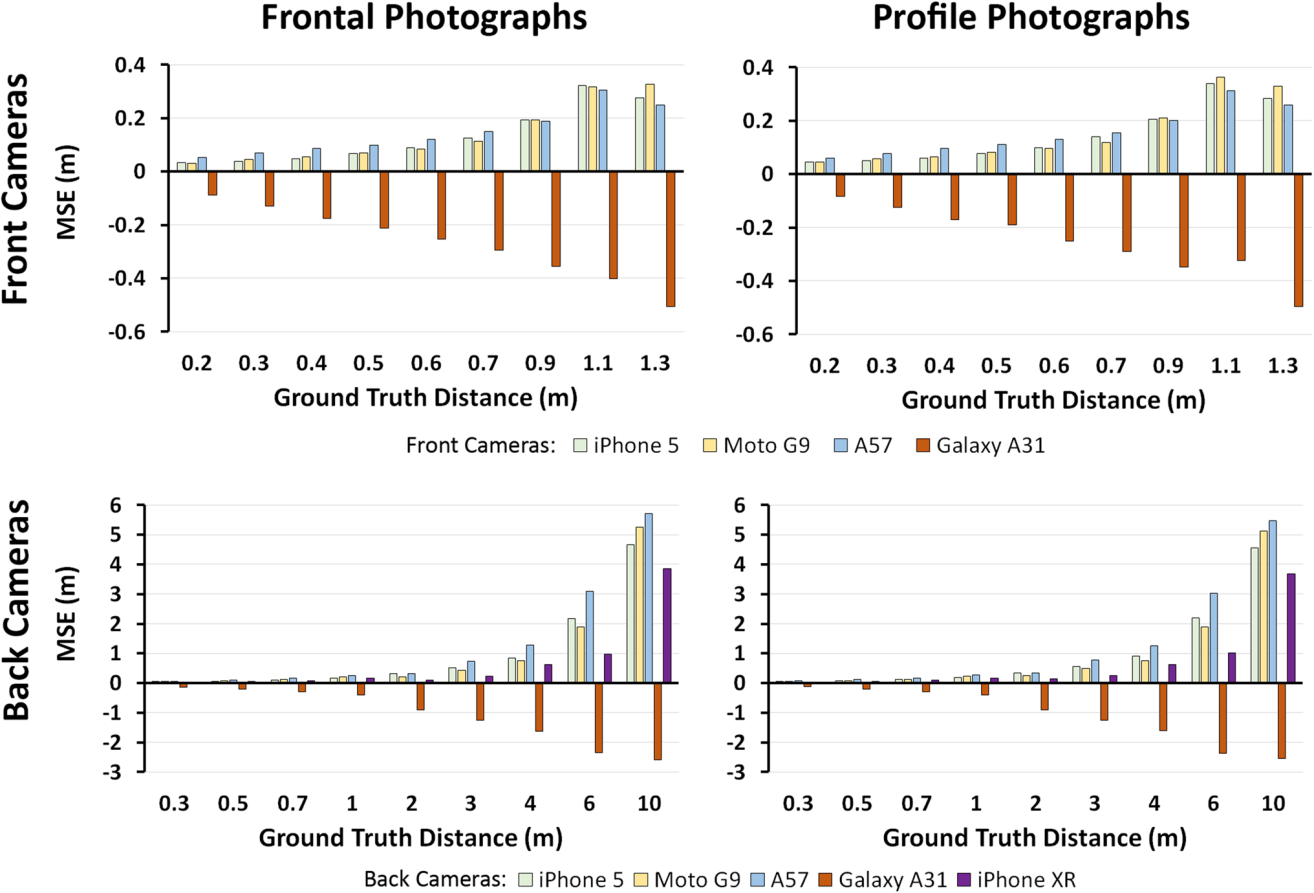
Mean signed error of *PerspectiveX* FD estimations of frontal and profile photographs taken using different smartphone cameras

For smartphone images, *PerspectiveX* estimations were considerably less accurate compared to the DSLR images (see Fig. 7 for MSE and Fig. S10-S12 for additional error metrics). For example, MAE% was 26% higher for the smartphones (Fig. S11). It should additionally be noted here that because the smartphone front cameras were tested at vastly different distances than the DSLRs (often much shorter FDs), the MSE and MAE are not directly comparable between the smartphones and the DSLRs (Fig. 7 and Fig. S10). As expected, the MSE increased as FD increased for three of the four smartphone front cameras, with little differences between the cameras (Fig. 7). The exception was the Samsung^®^ Galaxy A31, which consistently underestimated the FD, with saliently higher absolute error than the other front cameras at all distances (Fig. 7 and Fig. S10-S12).

The back cameras of the smartphones included FDs that were identical to the DSLR cameras, with MSE, MAE and MAE% errors of 570 mm, 1015 mm and 26%. Following the same trends seen in DSLR FD estimates, higher MAE was seen in profile photographs compared to frontal, with the error increasing with FD. Again, the Samsung^®^ Galaxy A31 showed a departure from the trends of the other smartphones, substantially underestimating the ground truth at every FD (Fig. 7).

The MAE% trends evident for front smartphone cameras were similar between their front and back componentry, with high error at the lowest distance, decreasing in mid-range FD to about 1.1 m, and thereafter increasing again (Fig. S11). The exception, the Galaxy A31 front camera, showed relatively stable MAE% across all FD ranges, but the error was of much larger magnitude (Fig. S11). The back camera MAE% slowly increased from 0.3 to 0.7 m, before dropping then rapidly increasing to a peak at 10 m, with the exception of the Samsung^®^ Galaxy A31 which was steady until 6 m, where error decreased (Fig. S11). There was no tested distance in which any of the smartphone front cameras were consistently accurate enough to be within the 1% facial height difference limit, whereas specific back cameras produced results below the threshold in the range of 2–10 m, but particularly more commonly for the longer distances above 6 m (Table S9).

## General discussion

This study provides the first sizable test of *PerspectiveX* on profile face images (*n* = 1709) using DSLR cameras and provides the first verification tests of FD estimates from smartphones. The results indicate that *PerspectiveX* (and its profile-view extension) work for both frontal and profile view images, respectively, as acquired on DSLR cameras and that the FD estimations errors are minimised in the 2–6 m ground truth range—where faces in the photographs hold good resolution. The data suggest that in craniofacial identification casework investigating authorities who liaise with families should seek facial photographs in the mid-FD range, i.e. not be taken too close or too far away to the camera and ideally between 2 and 3 m. Both frontal and partial profile or profile views should be obtained. The higher FD errors for smartphone images, than DSLR images, clearly indicate that DSLR images should be preferred for use in craniofacial superimpositions. In some cases, where DSLR images are not available, back camera smartphone images may be able to be used with acceptable degrees of error, but it depends heavily on what FD of the photograph is available for the superimposition.

Prior to conducting any superimposition, the FD error for the make of camera and lens used to acquire the image should be separately tested on an equivalent camera body/lens/smartphone in advance of casework to ensure the *PerspectiveX* method can be used for the particular make of camera at hand. This is easy to do since the electronic metadata records the camera and lens combinations used so that they can be sourced for testing. To validate a camera and lens, photographs of human subjects at an assortment of known whole integer FDs should be taken, similar to that conducted for this study. A review of the corresponding *PerspectiveX* estimates will demonstrate if the FD estimates are working for the camera prior to deployment in casework. If estimations are accurate, the camera system can be used. If the error is found to be too large, then, *PerspectiveX* should not be employed. The specific estimation of the FD used for the antemortem face photograph and subsequent replication for the skull will help prevent perspective mismatches in anatomical comparisons, thus improving superimposition comparisons (and reducing erroneous results).

### Study 1

For all photographs taken with DSLR cameras at distances of 3 m and more, FD estimates produced ≤ 1% error in facial height. For fixed prime lenses, 1% or less errors in facial height dimensions were observed at slightly shorter distances, i.e. from 2 m. As the resolution/size of the head is very poor/small at distances > 6 m when using typical portrait photography lenses (small to moderate focal length lenses), there is little value to considering FD estimation errors for FD larger than 6 m. Investigating authorities should, therefore, seek DSLR photographs taken with prime lenses at distances between 2 and 6 m, and ideally, we suggest 2–3 m. Only in the very specific and rare cases where extremely long focal length lenses are used, should photographs taken at distances over 6 m be used.

Across the full 1–10 m FD and for all cameras, lenses and focal lengths combined (15 total combinations), MSEs of 9.8% (frontal photographs), 10.8% (profile photographs) and 10.3% (both views combined) were observed. This is higher than the MSE (6.6%) reported across the same distance range by Stephan using only a Canon 6D fitted with a 100 mm prime lens for frontal only images [49]—also note here that Stephan tested FD estimates at each metre interval, whereas in this study, we only tested 6 intervals across the 10 m range. The MSE of this study (10.3%) is also several magnitudes higher than the error (3.8%) reported by Stephan and Armstrong [50], but again, note here that 1 m intervals were used across the 10 m FD range in [50], only frontal view images were used in [50] and only four total camera/lens/focal length combinations (all common to a Canon 6D camera body) were used in [50]. To increase comparability to the above studies by considering only the equivalent photographic view (frontal), camera, camera lens and focal length combinations of Stephan [49] and Armstrong and Stephan [50], then, the MSEs of this study are 4.4% and 8.8%, respectively. Subsequently, this study provides a slightly lower mean error value for the same camera combinations as [49] and just over double the error value of [50] for frontal view images alone.

The results indicate that the estimation of FD from DSLR *profile view photographs* [51] provides mean accuracies that are very close to *PerspectiveX* estimates from DSLR frontal view photographs. The mean errors from DSLR profile views taken using prime lenses in this larger sampled photographic study were also very similar for the original pilot test that also used a 2–6 m range, being 6.0% (this study) and 7.8% [51]. These data confirm the utility of FD estimation in superimposition casework *PerspectiveX* for profile view images and reconfirm *PerspectiveX’s* utility for frontal view photographs. As per Stephan and Armstrong’s [50] study, we also observed largest FD estimation errors for the maximum focal length setting of the variable zoom lenses. Subsequently, due to their inaccuracy, these variable zoom lenses should be avoided for craniofacial superimposition casework and favour awarded to images acquired from prime or fixed focal length lenses.

### Study 2

Overall, *PerspectiveX* was unable to accurately estimate FD of photograph taken with smartphone front and back cameras. There was no distance where estimates generated from any smartphone front camera were consistently within the tolerable range for 1% error in physiognomical face height. There were some longer FDs at which all estimates from specific back cameras fell within the 1% tolerance range (Table S9); however, due to the large field of views used by these cameras, the face recordings held poor resolution and limited utility for craniofacial superimposition.

Several factors may explain the poor estimation results for smartphones. It may in part be due to the difficulty in obtaining exact camera focal lengths and image receptor pixel sizes directly from smartphone manufacturers and instead relying on third-party websites or applications that may introduce inaccuracies. Additionally, it is possible undisclosed software manipulations of images occur between image capture and display/recording on the smartphone device that impact the FD estimate, such as undisclosed pixel binning procedures, because the software is proprietary in nature. It is intriguing that a similar method utilising iris size appears to be able to be successfully used for smartphone face images below 2 m FD [62], yet in this study, palpebral fissure lengths were generally unsuccessful. This may benefit from further attention and repeat studies using both *PerspectiveX* and MediaPipe Iris [62].

## Conclusions

The *PerspectiveX* extension to profile view images can be used to accurately estimate the FD from DSLR images of the face, but smartphone images cannot be used in frontal or profile view. For both frontal and profile photographs, this study shows that *PerspectiveX* works across multiple camera body/lens brands and models in the 2–6 m range. This suggests that the FD estimations provided by *PerspectiveX* may be generalisable to all DSLRs’ body and lens combinations on the market. Smartphone camera estimates were not accurate, so for now, these should be avoided in craniofacial superimposition casework when using *PerspectiveX*.

### Supplementary information

ESM 1 (PDF 399 kb)

ESM 2 (PDF 220 kb)

ESM 3 (PDF 217 kb)

ESM 4 (PDF 219 kb)

ESM 5 (PDF 213 kb)

ESM 6 (PDF 561 kb)

ESM 7 (PDF 597 kb)

ESM 8 (PDF 525 kb)

ESM 9 (PDF 138 kb)

ESM 10 (PDF 641 kb)

ESM 11 (PDF 538 kb)

ESM 12 (PDF 641 kb)

ESM 13 (PDF 274 kb)
