# Supplementary material for: Focus distance estimation from photographed faces: a test of PerspectiveX using 1709 frontal and profile photographs from DSLR and smartphone cameras
Source: Int J Legal Med. 2023 Sep 13;137(6):1907–20. doi: 10.1007/s00414-023-03078-y (PMC10567895; doi:10.1007/s00414-023-03078-y)
Supplement: Supplementary file 1 — (PDF 399 kb) [file 414_2023_3078_MOESM1_ESM.pdf]

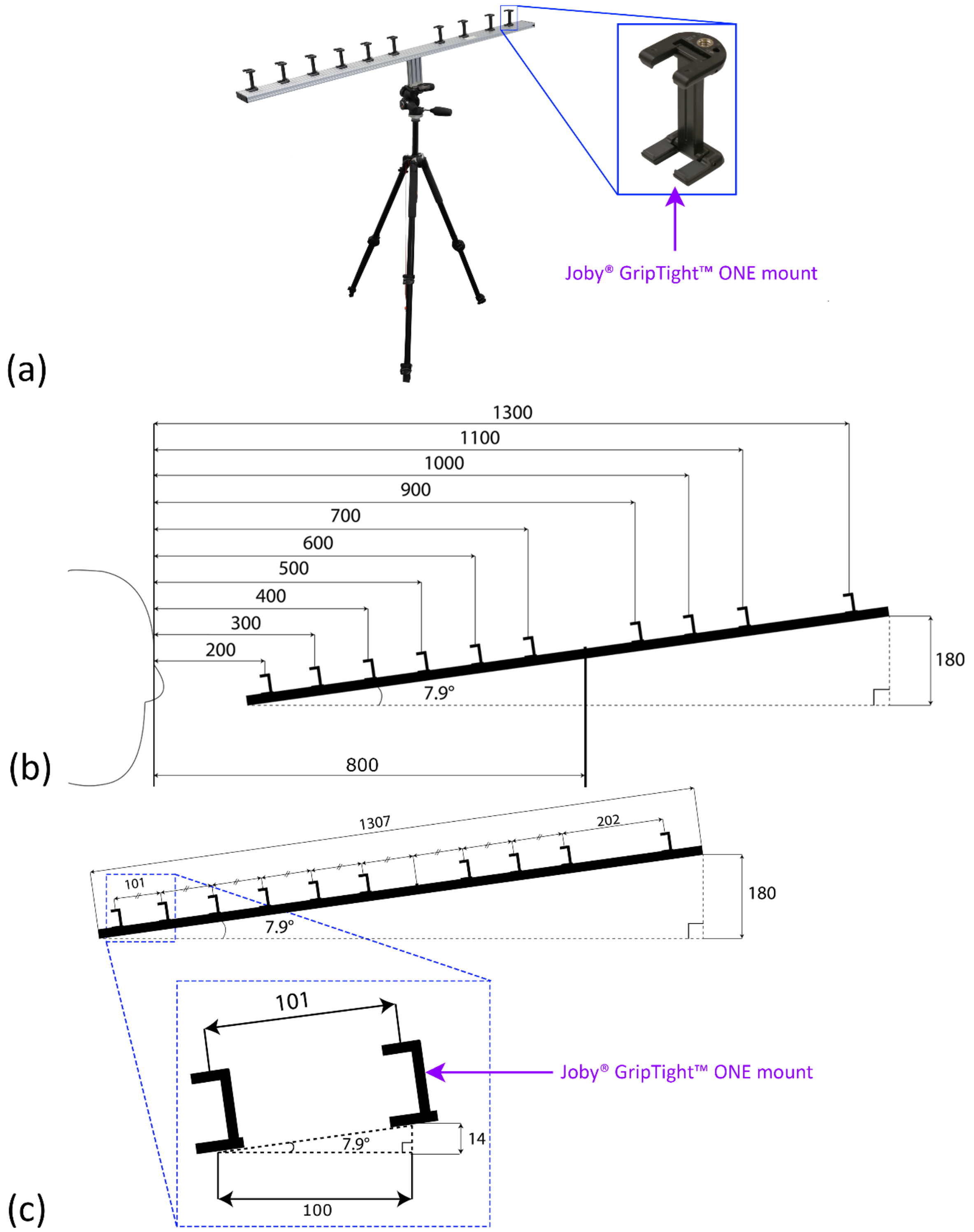


**Fig. S1** Photographic gantry used for smartphone photographs at focus distances ≤1.3 m. (a) Gantry with all mounts in upright position. The inset shows a single Joby® GripTight™ ONE mount. (b) Schematic showing the focus distances when cameras were placed in the mounts and the gantry in front of the participants' faces. (c) Mounts were placed 101 mm apart from each other with the

exception of the 7th and 10th mount which were 202 mm from the previous mount. The gantry was set at an angle of 7.9° to yield horizontal distance between the mounts of 100 mm. This led to teh focus distances seen in (b).

**Article title:** Focus Distance Estimation from Photographed Faces: A Test of PerspectiveX using 1,709 Frontal and Profile Photographs from DSLR and Smartphone Cameras

**Journal name:** International Journal of Legal Medicine

**Author names:** Sean S. Healy and Carl N. Stephan

**Affiliation:** Laboratory for Human Craniofacial and Skeletal Identification (HuCS-ID Lab)

**Corresponding author mail address:** sean.healy@uq.net.au
