# Supplementary material for: Focus distance estimation from photographed faces: a test of PerspectiveX using 1709 frontal and profile photographs from DSLR and smartphone cameras
Source: Int J Legal Med. 2023 Sep 13;137(6):1907–20. doi: 10.1007/s00414-023-03078-y (PMC10567895; doi:10.1007/s00414-023-03078-y)
Supplement: Supplementary file 3 — (PDF 217 kb) [file 414_2023_3078_MOESM3_ESM.pdf]

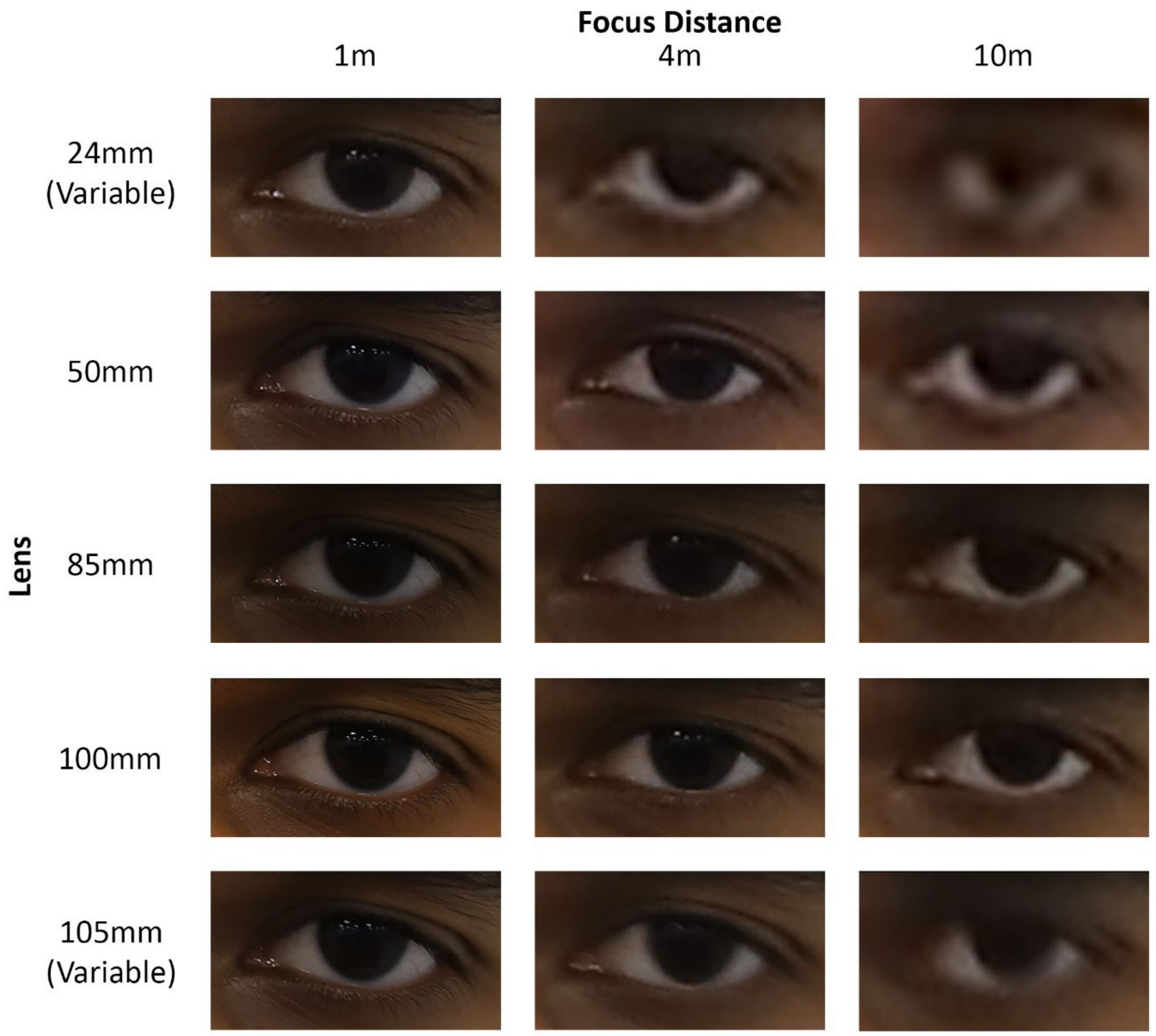


**Fig. S3** Example palpebral fissure recorded at different focus distances and with different cameras lenses for the same subject photographed with the same camera body (Canon® EOS 6D Mark II). For comparability, all images have been fixed-aspect ratio scaled to the same palpebral fissure length size (which is identical to that used for Fig. 4). Note: this enlargement has resulted in resampling of the lower resolution images to 300 pixels per inch, adding some smoothing to what were originally more pixelated images.

**Article title:** Focus Distance Estimation from Photographed Faces: A Test of PerspectiveX using 1,709 Frontal and Profile Photographs from DSLR and Smartphone Cameras

**Journal name:** International Journal of Legal Medicine

**Author names:** Sean S. Healy and Carl N. Stephan

**Affiliation:** Laboratory for Human Craniofacial and Skeletal Identification (HuCS-ID Lab)

**Corresponding author mail address:** sean.healy@uq.net.au
