# Supplementary material for: Focus distance estimation from photographed faces: a test of PerspectiveX using 1709 frontal and profile photographs from DSLR and smartphone cameras
Source: Int J Legal Med. 2023 Sep 13;137(6):1907–20. doi: 10.1007/s00414-023-03078-y (PMC10567895; doi:10.1007/s00414-023-03078-y)
Supplement: Supplementary file 6 — (PDF 561 kb) [file 414_2023_3078_MOESM6_ESM.pdf]

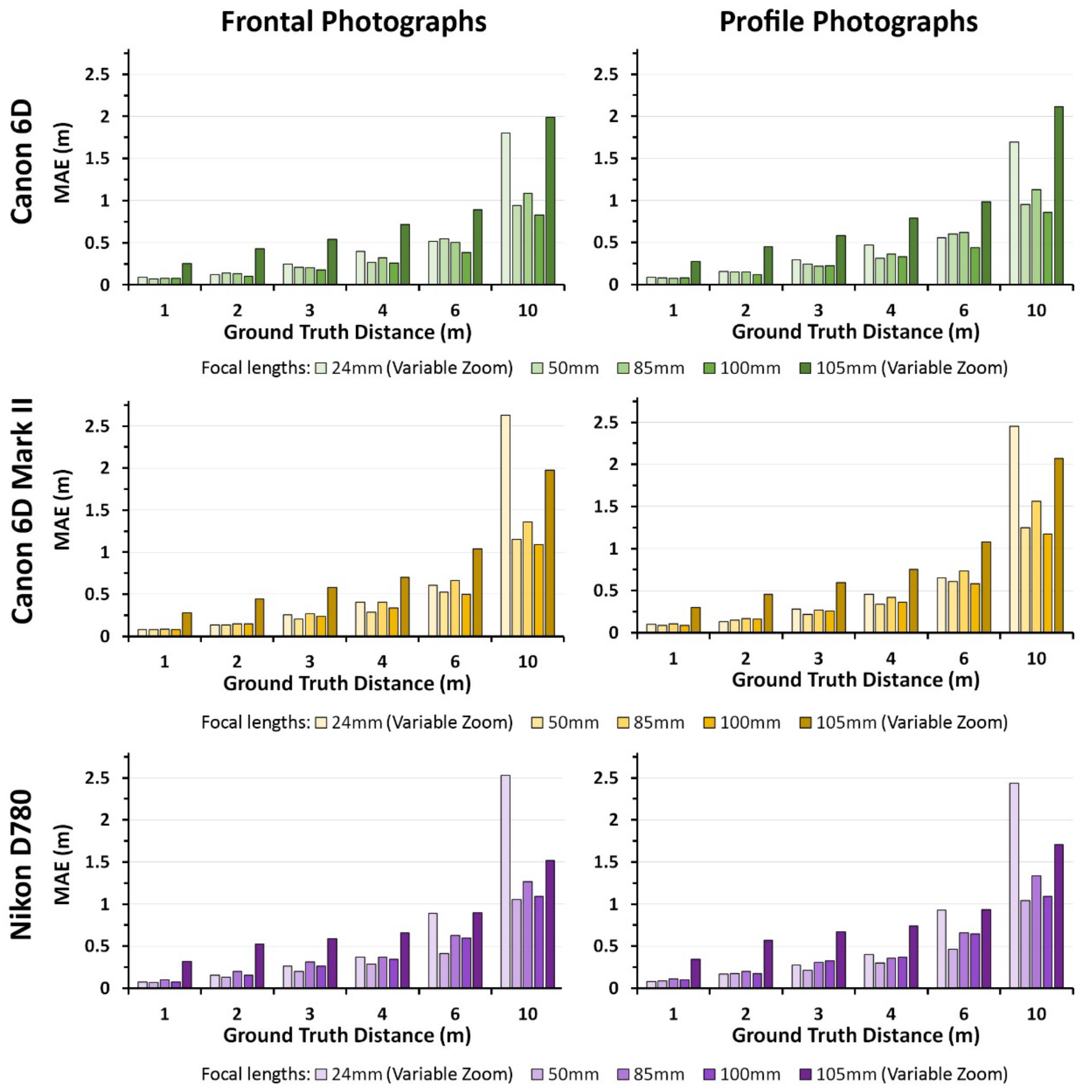

Fig. S6 Mean absolute error of *PerspectiveX* focus distance estimations of frontal and profile photographs taken using different DSLR cameras and lenses.

Article title: Focus Distance Estimation from Photographed Faces: A Test of PerspectiveX using 1,709 Frontal and Profile Photographs from DSLR and Smartphone Cameras

Journal name: International Journal of Legal Medicine

Author names: Sean S. Healy and Carl N. Stephan

Affiliation: Laboratory for Human Craniofacial and Skeletal Identification (HuCS-ID Lab)

Corresponding author mail address: sean.healy@uq.net.au
