# Supplementary material for: Focus distance estimation from photographed faces: a test of PerspectiveX using 1709 frontal and profile photographs from DSLR and smartphone cameras
Source: Int J Legal Med. 2023 Sep 13;137(6):1907–20. doi: 10.1007/s00414-023-03078-y (PMC10567895; doi:10.1007/s00414-023-03078-y)
Supplement: Supplementary file 9 — (PDF 138 kb) [file 414_2023_3078_MOESM9_ESM.pdf]

Camera Body/Phone	Lens Type/Position	Focal Length (mm)	Photograph View	Focus Distance Range Yielding ≤1% Perspective Distortion Mismatch (m)
Canon® 6D	Prime	50	Frontal	2-10
			Profile	
		85	Frontal	
			Profile	
		100	Frontal	
			Profile	
	Variable Zoom	24	Frontal	
			Profile	
		105	Frontal	3-10
			Profile	
Canon® EOS 6D Mark II	Prime	50	Frontal	2-10
			Profile	
		85	Frontal	
			Profile	
		100	Frontal	
			Profile	
	Variable Zoom	24	Frontal	
			Profile	
		105	Frontal	3-10
			Profile	
Nikon® D780	Prime	50	Frontal	2-10
			Profile	
		85	Frontal	
			Profile	
		105	Frontal	
			Profile	
	Variable Zoom	24	Frontal	
			Profile	
		120	Frontal	3-10
			Profile	
Apple® iPhone™ 5	Front	2.18	Frontal	NA
			Profile	
	Back	4.15	Frontal	4-10
			Profile	10
Apple® iPhone™ XR	Back	4.25	Frontal	2-10
			Profile	
Motorola® Moto G9 Plus	Front	3.78	Frontal	NA
			Profile	
	Back	5.53	Frontal	3-10
			Profile	
Oppo® A57	Front	3.57	Frontal	NA
			Profile	
	Back	3.46	Frontal	4-10
			Profile	6-10
Samsung® Galaxy A31	Front	3.8	Frontal	NA
			Profile	
	Back	4.6	Frontal	
			Profile	

Fig. S9 Results for DSLR camera bodies and smartphone cameras where focus distance estimates did not generate perspective distortion differences above 1%.

Article title: Focus Distance Estimation from Photographed Faces: A Test of PerspectiveX using 1,709 Frontal and Profile Photographs from DSLR and Smartphone Cameras

Journal name: International Journal of Legal Medicine

Author names: Sean S. Healy and Carl N. Stephan

Affiliation: Laboratory for Human Craniofacial and Skeletal Identification (HuCS-ID Lab)

Corresponding author mail address: sean.healy@uq.net.au
